# Parametric Analysis of Epoxy/Crumb Rubber Composite by Using Taguchi—GRA Hybrid Technique

**DOI:** 10.3390/polym13193441

**Published:** 2021-10-07

**Authors:** Kiran Shahapurkar, Venkatesh Chenrayan, Belay Brehane Tesfamarium, Manzoore Elahi M. Soudagar, Nazia Hossain, Ali A. Rajhi, Sagr Alamri, Ibrahim M. Alarifi, Pavan Shahapurkar, M. A. Mujtaba, M. C. Kiran, Gulam Mohammed Sayeed Ahmed

**Affiliations:** 1 School of Mechanical, Chemical and Materials Engineering, Adama Science and Technology University, Adama 1888, Ethiopia; kiranhs1588@astu.edu.et (K.S.); venkymech0607@gmail.com (V.C.); bellove22@gmail.com (B.B.T.); 2 Department of Mechanical Engineering, School of Technology, Glocal University, Delhi-Yamunotri Marg, SH-57, Mirzapur Pole, Saharanpur District, Uttar Pradesh 247121, India; me.soudagar@gmail.com; 3 School of Engineering, RMIT University, Melbourne, VIC 3001, Australia; 4 Department of Mechanical Engineering, College of Engineering, King Khalid University, Abha 61421, Saudi Arabia; arajhi@kku.edu.sa (A.A.R.); salamri@kku.edu.sa (S.A.); 5 Department of Mechanical and Industrial Engineering, College of Engineering, Majmaah University, Al-Majmaah, Riyadh 11952, Saudi Arabia; i.alarifi@mu.edu.sa; 6 Applied and Theoretical Mechanics Laboratory, Discipline of Mechanical Engineering, Indian Institute of Technology, Indore 453552, India; shahapurkarpa1@gmail.com; 7 Department of Mechanical Engineering, Faculty of Engineering, University of Malaya, Kuala Lumpur 50603, Malaysia; m.mujtaba@uet.edu.pk; 8 Department of Mechanical Engineering, Nitte Meenakshi Institute of Technology, Bangalore 560064, India; 20.kiranmc@gmail.com; 9 Department of Mechanical Design and Manufacturing Engineering, Adama Science and Technology University, Adama 1888, Ethiopia; drgmsa786@gmail.com; 10 Center of Excellence (COE) for Advanced Manufacturing Engineering, Department of Mechanical Design and Manufacturing Engineering, Adama Science and Technology University, Adama 1888, Ethiopia

**Keywords:** polymer composites, crumb rubber, erosion rate, erosion efficiency, grey relational grade

## Abstract

Effect of parameters affecting solid particle erosion of crumb rubber epoxy composite is investigated. Five important process parameters—impact velocity, impingement angle, standoff distance, erodent size, and crumb rubber content—are taken into consideration. Erosion rate and erosion efficiency are included as the chief objectives. The Taguchi coupled gray relational analysis type statistical model is implemented to study interaction, parameters’ effect on responses, and optimized parameters. ANOVA and regression model affirmed impingement angle and crumb rubber content play a significant role to minimize the erosion. Validity of the proposed model is justified with the standard probability plot and R^2^ value. A confirmation experiment conducted with A_2_B_2_C_3_D_3_E_3_ condition registers noticeable enhancement in GRG to the tune of 0.0893.

## 1. Introduction

Composite materials are realized by amalgamating two or more constituents differing in physical and chemical form and are insoluble in each other. Composite materials are formed to benefit from the superior properties of constituents without compromising on the desired properties. Low density, good resistance to corrosion, fabrication ease, and low cost of engineered polymer composites make them desirable for many applications [1,2,3]. In particulate reinforced polymer composites [4], the matrix is the load-bearing constituent and facilitates load transfer, whereas particulate filler assists in improving the functional properties of composites [5,6,7,8,9]. These composites are extensively used in airplane parts, marine, transportation, defense, and power sectors. In addition, to meet the specific requirement of envisaged applications, polymer composites are required to function under a severe environment of dust and solid particles impingement. Therefore, the tribological response of polymer composites becomes an important aspect to assess the composites. In particular, polymer composites’ solid particle erosion behavior needs to be studied in more detail as these composites are often subjected to severe environments wherein solid particle impingements lead to deterioration of material properties.

Solid particle erosion occurs due to repeated impacts of eroding material on the target material, leading to material loss from the target. Such a phenomenon occurs in several machinery like gas turbine blades of an aircraft, pump impellers in processing equipment, windscreens, space applications parts of missiles, and so on. Mechanical strength does not always ensure erosion resistance, and therefore a detailed investigation of the material characteristics is essential for minimization of erosion. Loss of material due to solid particle impingements is dependent on several interrelated factors. Experimental condition, properties of the target specimen, and size and shape of erodent material are the main factors affecting erosion response of composites. In addition to these factors, loss of material due to solid particle erosion is very complex. It involves many multifaceted processes like micro-plowing, micro-cutting, glazing, platelet formation, fragmentation, fatigue, and so on.

The addition of fillers in enhancing the performance of polymer composites for tribological applications has gained significant interest in the recent past owing to their favorable properties for mechanical strength enhancement and cost reduction of components. Generally, filler materials are categorized as organic, inorganic, and metallic and are available in micro and nano sizes. The inclusion of filler materials is mainly intended to enhance specific properties and cost-saving. Crumb rubber is one such filler material derived from waste and nondisposable tires. Crumb rubber material is comprised of 56% carbon, 19% oxygen, 8% sulfur, 6% calcium, and trace quantities of aluminum, silicon, sulfur, titanium, iron, and zinc [10]. Disposal of waste tires is a challenging task, and discovering new ways to overcome this is the need of the hour. The use of crumb rubber material in synthesizing polymer composites can benefit both environment and economy. Crumb rubber is used to develop composites focused on abrasion resistance [11] and different loading conditions [7,9,12,13,14,15,16,17,18,19]. The optimal set of parameters to minimize the erosion rate in polyester composite reinforced with E-glass fiber were declared as 58m/s velocity, 53% fiber loading, 780µm erodent size, and 181 mm standoff distance [20]. Therefore, it is clear that the optimal parameters differ from one material to another to yield an effective erosion rate. The order of significance to minimize the erosion rate of Alumina–GF–Polyester hybrid composite was reported as Alumina percentage, impingement angle, erodent size, and velocity [21]. Taguchi’s design of experiment method implemented for the hybrid composite made of glass fiber and epoxy reinforced with Al_2_O_3_+ SiO_2_ revealed that the maximum erosion rate was noticed at 30° impingement angle [22]. The experimental statistical analysis performed to study the effect of parameters over the erosion rate for different polymer composite with different reinforcement invariably recognized the importance of percentage of reinforcement, impingement angle, and impact velocity [23].

The behavior of materials was characterized as either ductile or brittle based on the rate of erosion attained with different impinging angles. The inclination of the sample surface to the line of erodent particles is called impact angle [16]. The behavior of the samples is considered ductile if the maximum erosion rate is observed between 15 and 30°, while the behavior is deemed to be brittle for the samples wherein the maximum erosion rate occurs at 90. Further, the behavior is considered to semi-ductile if erosion occurs occurs between 45 and 60°. However, the classification of the materials cannot be regarded as absolute because reverse trends were observed with many materials when eroded with erodent particles varying in size [24,25,26,27,28]. Erosion experiments on different metals, polymers, and ceramic materials also revealed that the hardness of erodent could not sufficiently describe the responses observed [29]. Therefore, a combination of hardness and fracture toughness are used to associate the erosion of metals, polymers, and ceramics [23,29,30,31]. Moreover, hardness only depicts the volume dislodged by every impinging erodent and not the exact erosion. To overcome these shortcomings, Sundararajan et al. [32] proposed a parameter called erosion efficiency to assess the prominent erosion mechanisms considering the impact velocity, hardness, and density of erodent particles. 

This research attempts to study and analyze the effect of multiple parameters on the solid particle erosion response of crumb rubber–filled epoxy novel composites using Taguchi coupled gray relational analysis. Erosion rate and erosion efficiency are considered as responses by extending the investigation as the multi-response problem. None of the literature reported the optimal parametric analysis of polyester composites erosion rate and efficiency as the key responses. Parametric optimizations of numerous engineering problems are performed using Taguchi’s method, including solid particle erosion of composites [33,34,35,36].

## 2. Materials and Methods

### 2.1. Materials

Crumb rubber particles procured from Arihant Chemicals, Delhi, India, are reinforced in LAPOX L-12 epoxy resin and K6 hardener supplied by Atul Industries, Gujarat, India, to prepare particulate polymer composites. Crumb rubber particles have a density of 1451 kg/m^3^ and modulus of 2600 MPa, while LAPOX L-12 epoxy has a density of 1192 kg/m^3^ and modulus in the range of 30–40 GPa. Crumb rubber particles have a average particle size of 182.24 μm.

### 2.2. Sample Preparation

**Figure 1:**
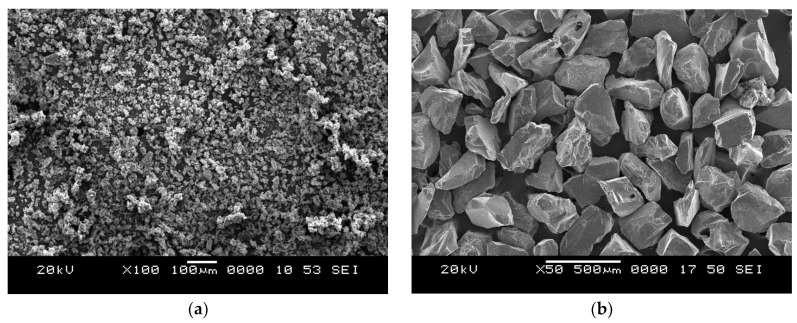
Scanning electron micrographs of (**a**) Crumb rubber and (**b**) Silicon carbide erodent.

Composite slabs are prepared by the conventional open mold casting method. Composites with three varying compositions of crumb rubber (10, 20, and 30 vol.%) are prepared. Composites slabs are prepared by mixing desired quantity of constituents in a glass beaker using glass rods to achieve a uniform and consistent slurry. Further, a hardener in the ratio of 10 wt.% of epoxy resin is added to the slurry to start the polymerization process. The slurry is finally poured in an aluminum mold coated with a silicone releasing agent for easy removal of slabs. Castings are cured for 24 h at ambient temperature and trimmed to suitable dimensions as per ASTM standards. Scanning electron micrograph of crumb rubber particle and silicon carbide erodent is depicted in Figure 1a,b, respectively. The shape of erodent particles is irregular in shape and intended to remove maximum material from the target surface.

### 2.3. Erosion Test Rig

**Figure 2:**
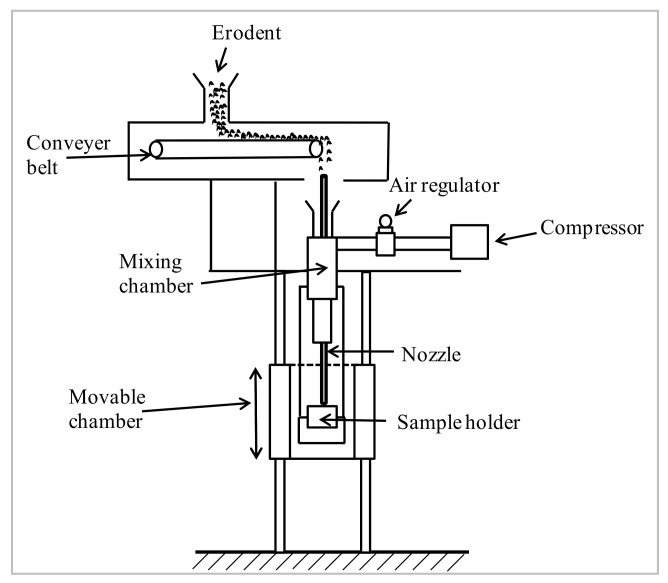
Schematic representation of erosion test rig.

Schematic representation of erosion test setup is depicted in Figure 2. The setup confirms to ASTM G76 standard and is capable of reproducing repetitive erosive conditions to assess the erosive resistance of samples. The test rig comprises of an air compressor, mixing chamber (air and erodent particles), and accelerating compartment. Erodent particles are fed through the conveyor belt to the mixing chamber in which dry compressed air is supplied. Compressed erodent particles influence on the target samples with the aid of an accelerating chamber and convergent nozzle of 1.5 mm diameter. Samples can be held at different angles concerning the particles’ impingement. Standoff distance is the distance between the tip of the nozzle and specimen holder. The standoff distance can be varied by moving the specimen holder with respect to the fixed nozzle. The velocity of the erodent particles is determined using the standard double-disc method [20]. Samples are cleaned in acetone and weighed to an accuracy of ±0.001 mg using a precise electronic balance before the test. Weight loss before and after the tests are recorded for observing the erosion rate. The process as mentioned earlier is repeated until the erosion rate achieves a stable state called steady-state erosion rate.

### 2.4. Erosion Efficiency

Erosion efficiency is an effective parameter to evaluate the behavior of the material [37]. It considers all the parameters associated with the erosion, and therefore it serves as an effective validation tool to support the experimental or analytical results.

(1) $$Erosion\;Efficiency=\frac{2EH}{\delta v^{2Sin^{2}\alpha }}$$

where

*E* is the erosion rate;
*H* is the hardness of the eroding material;
*δ* is the density of eroding material;
v  is the impact velocity of erodent particles;
*α* is the angle between the sample surface and line of erodent impact.

### 2.5. Experimental Design and Procedure

Usually, the design of the experiment method is used to know the degree of significance of each control parameter over the uncontrolled responses and to optimize the effect of parameters over the responses considered. For the current research work, five process variables were considered to influence the erosion rate and erosion efficiency: velocity, angle of impingement, percentage of composition of crumb rubber, standoff distance, and size of the erodent. The value and the levels of each parameter were assigned based on previous literature [4,20,38], as shown in Table 1. Taguchi’s L27 orthogonal array was selected to deal with five parameters and two responses unless otherwise, more experiments were needed, which are not economical.

### 2.6. Gray Relational Analysis

Gray relational analysis is a famous statistical tool to converge the multi-objective problems into a single objective. The combined single gray relation grade was arrived between 0 and 1 for each experiment by analyzing and normalizing the observed responses with a structured statistical way.

Step 1:

The signal to noise (*S/N* ratio) was employed to assess the quality characteristics based on the Taguchi method [37]. Based on the requirements of the effect of responses like smaller, larger, and nominal, few prescribed relations are available [39,40] as per the Taguchi method. The responses of the present study are erosion rate and erosion efficiency, which required being as minimum as possible. The following relation was used to determine the *S/N* ratio, based on minimum the better principle.

(2) $$\frac{S}{N}Ratio=-10\;\log\left(\frac{1}{n}\overset{n}{\underset{j=1}{\sum \;y_{j}^{2}}}\right)$$

Step 2:

Normalizing the observed experimental data is mandatory since the data were recorded in different sources and different units. Normalization is a process of reducing the empirical experimental data into a range of 0 to 1 (0 ≤ Z_ij_ ≤ 1). Normalization of data was carried out with the following relation.

(3) $$Z_{\mathrm{ij}}=\frac{Max\left(y_{ij},\;i=1,2,3,\ldots m\right)-y_{ij}}{Max\left(y_{ij},\;i=1,2,3,\ldots m\right)-Min\left(y_{ij},\;i=1,2,3,\ldots m\right)}$$

Step 3:

In the computation of gray relational coefficient, the relationship between optimal and normalized experimental results was exposed with the help of the gray relational coefficient to achieve the performance characteristic. The following equation calculated the gray relation coefficient for each experiment response.

(4) $$GC_{ij\;}=\frac{{∆}_{min}+\lambda \left({∆}_{max}\right)}{{∆}_{ij}+\lambda \left({∆}_{max}\right)}$$

where, ${∆}_{ij}$ is the deviation sequence, the value ${∆}_{min}=0\;,\;{∆}_{max}=1\;\mathrm{and}\;\mathrm{the}\;\mathrm{value}\;\mathrm{of}\;“\lambda ”\;\mathrm{is}\;\mathrm{usually}\;\mathrm{considered}\;\mathrm{as}\;0.5,$ to provide equal weightage to all.

Step 4:

The gray relational grade is an indicator of geometric similarity between reference series and gray system. The gray relational grade is a simple average of the gray relational coefficients of each sequence, which the following equation can manipulate.

(5) $$GC_{ij\;}=\frac{1}{m}\overset{m}{\underset{i=1}{\sum }}GC_{j}$$

## 3. Results and Discussions

The sequence of the experiment and observed response values for each experiment are shown in Table 2. The computed S/N ratios, Normalized S/N ratios, Gray Relational Coefficient, and Gray Grade are as shown in Table 3.

### 3.1. Effect of Velocity

The impact velocity of the erodent particles on the specimen plays a considerable effect on the erosion rate of composites. In the present investigation, it is observed that an impact velocity of 45 m/s depicts a lower erosion rate than others. Low-velocity impact induces insufficient stresses for plastic deformation, and erosion occurs due to glazing, whereas higher velocity erodes the specimen material plastically due to repetitive plastic deformation [41,42]. Impact velocities of 60 m/s depict high erosion compared with 45 m/s, which may be attributed to melting the affected surface and thereby reducing the erosion rate [41].

### 3.2. Effect of Impingement Angle

The impingement angle of the specimen to the impact of erodent particles also plays a crucial role in the erosion rates of the composites. Impingement angles are usually varied between 15 and 90° to study the response of composites to erosion. Low impingement angles (<30°) show ductile behavior, while impingement angles near to incidence reveal brittle behavior, and impingement angles between 45 and 60° show semi-ductile or semi-brittle behavior [6]. Based on the analysis, the semi-ductile response is shown by the composites. Reinforcing relatively softer crumb rubber particles in the brittle epoxy matrix has induced semi-ductile behavior in the composites. Similar observations can be observed in Refs. [43,44,45,46].

### 3.3. Effect of Crumb Rubber

The addition of a filler is intended to enhance the erosion resistance of the composites. In the present study, the higher content of crumb rubber particles reinforcement in the epoxy matrix shows higher resistance to erosion. Replacing the hard and brittle epoxy matrix with relatively soft crumb rubber particles provides higher resistance to erosion, mainly attributed to the composite’s ability to sustain the impact of erodent particles.

### 3.4. Effect of Standoff Distance

Standoff distance is the distance between the tip of the nozzle and the target specimen. In the present study, 150 mm of standoff distance is recognized as the optimum distance to ensure the minimum erosion rate of composites.

### 3.5. Effect of Erodent Size

**Figure 3:**
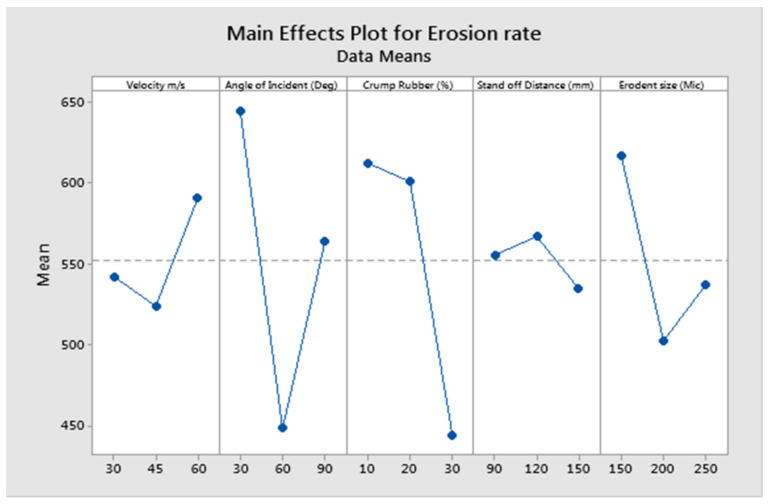
Effect of control factors on solid particle erosion.

The erosion rate is independent of erodent particle size beyond a critical value and lies between 100 and 200 μm [41,47,48]. Erosion tests performed with erodent particles in the range of 100 to 200 μm depict a higher erosion rate with the increase in the size of erodent particles [41]. However, above the critical value (>200 μm), erosion rates are seen to decrease owing to the limited amount of erodent particles impacting the target surface [16]. Silicon carbide erodent particles are irregular in shape intended to erode maximum material from the target. However, the lowest erosion rates are observed with 250 μm erodent particles in the present study. Analysis of the overall result concludes that factor combination of A_2_, B_2_, C_3_, D_3_, and E_3_ gives minimum erosion rate as evident from Figure 3. The statistical means of GRG were used to draw the main effects plot and largest mean value is considered to be the opimum level of parameter.

### 3.6. ANOVA (Analysis of Variance) Analysis

The individual degree of influence of each parameter over the responses was determined through ANOVA analysis. Table 4 shows the ANOVA analysis for erosion rate. The results confirm that the impingement angle becomes the most critical parameter to influence the erosion rate by ensuring 47.51% contribution and by fulfilling the statistical requirement of p-value less than 0.05. The percentage of crumb rubber scores second rank in the contribution list. The rest of the parameters like velocity and crumb rubber composition are claiming meager significance. It is observed that the size of the rodent also has a considerable importance.

Similar ANOVA analysis for the erosion efficiency is shown in Table 5. The results reveal that the velocity becomes the most significant parameter to influence the erosion efficiency. The rest of the parameters are considered to be less effective.

### 3.7. Mathematical Model

The relation between the parameters and the responses was investigated by developing a linear regression equation. The regression coefficient value R^2^ is in good agreement with the adjusted R^2^ value. Both the coefficient values are nearer to the unity, which acknowledges the excellent relationship of the parameters with the responses. The mathematical developed for Erosion rate is

(6) R=552.4−28.5 A+103.9B+108.3C−17.7D+15.1E

where, the notations are: *A*—Velocity, *B*—Angle of impingement, *C*—Crumb rubber percentage, *D*—Standoff distance, and *E*—Erodent size. The above equation highlights that the erosion rate is directly proportional to the parameters like angle of impingement, crumb rubber, and erodent size by securing the + ve signs. On the other hand, the parameters velocity and standoff distance do not have much influence over the responses, which could be understood by their–ve signs [49].

The equation developed for response erosion efficiency given below also shows the excellent relation of parameters with responses. The degree of relationship is validated by the closest value of the regression coefficients R^2^ and adjusted R^2^ with the value of one. The regression equation for erosion efficiency is given by

**Figure 4:**
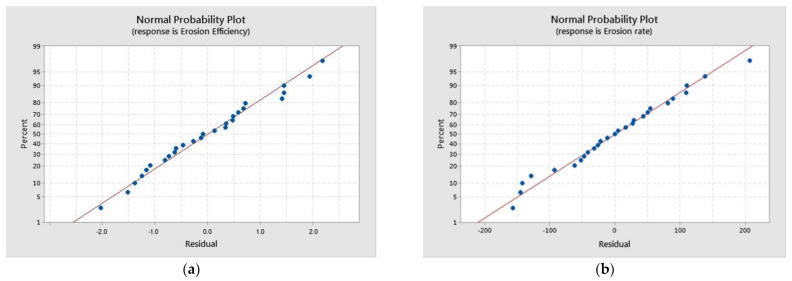
Standard probability plot for (**a**) Erosion efficiency and (**b**) Erosion rate.

(7) EF=14.724+5.24 A+2.904B−193.8 C−1.978 D−0.236 E

where the notations are: *A*—Velocity, *B*—Angle of impingement, *C*—Crumb rubber percentage, *D*—Standoff distance, and E—Erodent size. The equation reveals the nature of the relation of the parameters with the responses. The velocity and angle of impingement are directly proportional to the responses, which is acknowledged by their + ve sign. However, the parameters crumb rubber, standoff distance, and erodent size are not directly proportional to the responses, confirmed with their–ve sign. Figure 4 shows the average probability for erosion rate and efficiency, which acknowledges that most of the points lie between the zero standardized region. Hence, the recommendation of the proposed model is very precise and can be used for customized variations of the parameters to achieve the optimized erosion rate and erosion efficiency in the future.

### 3.8. Verification of the Optimal Parameters

Verification of optimal parameters has to be conducted to justify the model and to validate the proposed mathematical model. Hence, a confirmation experiment was conducted by following the optimal parameters, and GRG was subsequently manipulated for the optimal level. A predicated GRG for the recommended optimal level of parameters A_2_B_2_C_3_D_3_E_3_ was calculated with the following equation.

(8) $$a_{Pre}=a_{m}+\overset{n}{\underset{i=1}{\sum }}\left(a_{0}-a_{m}\right)$$

where *a*_*Pre*_ is the predicted gray relation grade, *a*_*m*_ is the mean average of the gray grades, *a*_0_ is the average gray grade of the optimal level of the parameters (A_1_B_2_C_2_), and ‘*n*’ is the number of essential factors considered from the response table.

**Figure 5:**
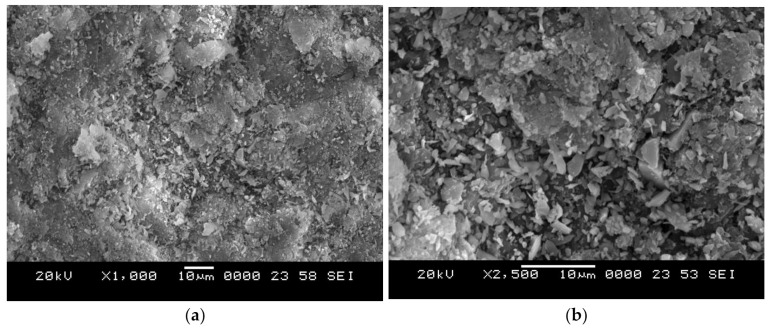
Scanning electron micrograph of the sample tested for A_2_B_2_C_3_D_3_E_3_ condition at (**a**) lower and (**b**) higher magnification.

Table 6 shows the results of the confirmation experiments, which notifies the improvement in GRG to 0.0893 during experimental response observation. This again validates that the proposed gray relational model can be appropriate to optimize the erosion responses with variable materials. Figure 5 presents the micrograph of the sample tested for A_2_B_2_C_3_D_3_E_3_ condition. It can be observed clearly that the crumb rubber particles effectively resist the erosion and the wear observed is very minimal at optimized parametric conditions.

## 4. Conclusions

Through this research, a novel epoxy-based polymer matrix reinforced with different proportions of crumb rubber was developed. The erosion rate and erosion efficiency were experimentally evaluated through an erosion test rig. Twenty-seven numbers of experiments were conducted as per Taguchi’s L27 orthogonal array. The various conclusions and observations obtained in this study are listed below. The velocity of 45 m/s, impingement angle of 60°, 30% crumb rubber proportion, 150 mm distance of standoff distance, and 250 µm of erodent size were found to be the optimal erosion parameters to achieve the best erosion rate and erosion efficiency. As far as erosion rate is concerned, the regression model and ANOVA results acknowledge that an angle of impingement is supposed to be a chief influencing parameter followed by a percentage of crumb rubber composition. The erodent size plays the least significant role in influencing the erosion rate. The velocity becomes the most significant parameter to affect the erosion efficiency, followed by meager importance in influencing erosion efficiency.

The value of R^2^ from ANOVA analysis for both the responses is converging to unity. The standard probability plot for both responses shows that most points lie in the zero residual regions. These two findings uphold the highest suitability of the proposed model to the present study. GRG evaluated for the confirmation experiment by following the optimal parameter level of A_2_B_2_C_3_D_3_E_3_ shows considerable improvement over the predicted one. The regression equation developed for the erosion rate confirms the direct proportionality of the percentage of crumb rubber composition with the erosion rate. In turn, the ANOVA results also acknowledge its degree of significance to the second rank. The statistical study reveals that the newly developed crumb rubber reinforced epoxy polymer matrix composite is a good erosion-resistant composite.

## Figures and Tables

**Table 1:** Erosion parameters and their levels.

S. No.	Parameters	Symbol	Unit	Level		
				1	2	3
1	Velocity	A	m/s	30	45	60
2	Angle of Impingement	B	Degree	30	60	90
3	Crumb Rubber Composition	C	%	10	20	30
4	Standoff Distance	D	mm	90	120	150
5	Erodent size	E	Micron	150	200	250

**Table 2:** Experimental design and response observation.

S.No.	Velocity (m/s)	Angle (°)	Crumb Rubber (%)	Standoff Distance (mm)	Erodent Size (μm)	Erodent Rate (mg/g)	Erodent Efficiency (%)
1	30	30	10	90	150	730.86	36.27
2	30	30	20	120	200	554.93	27.76
3	30	30	30	150	250	543.50	24.67
4	30	60	10	90	200	409.91	20.34
5	30	60	20	120	250	624.97	31.27
6	30	60	30	150	150	330.15	16.64
7	30	90	10	90	250	682.85	33.90
8	30	90	20	120	150	536.63	26.84
9	30	90	30	150	200	466.78	23.52
10	45	30	10	150	150	752.16	12.44
11	45	30	20	90	200	825.14	13.76
12	45	30	30	120	250	454.93	9.32
13	45	60	10	150	200	412.67	6.82
14	45	60	20	90	250	414.20	5.63
15	45	60	30	120	150	407.57	6.85
16	45	90	10	150	250	433.94	7.18
17	45	90	20	90	150	678.96	11.32
18	45	90	30	120	200	435.89	12.36
19	60	30	10	120	150	932.00	11.57
20	60	30	20	150	200	507.61	6.34
21	60	30	30	90	250	501.38	7.13
22	60	60	10	120	200	489.09	6.07
23	60	60	20	150	250	612.82	7.66
24	60	60	30	90	150	435.07	5.49
25	60	90	10	120	250	666.81	8.27
26	60	90	20	150	150	752.39	9.41
27	60	90	30	90	200	421.32	9.09

**Table 3:** Gray relation coefficient with grade and their rank.

S. No.	S/N Ratio		Normalized S/N Ratio		Gray Relational Coefficient		Gray Relational Grade		CGRG	Rank
	(ER)	(EF)	(ER)	(EF)	(ER)	(EF)	(ER)	(EF)		
1	−42.96	−16.88	0.2235	0.0166	0.7765	0.9834	0.3917	0.3371	0.3644	27
2	−40.57	−14.56	0.4766	0.1525	0.5234	0.8475	0.4886	0.3711	0.4298	24
3	−40.39	−13.53	0.4957	0.2125	0.5043	0.7875	0.4979	0.3884	0.4431	21
4	−37.94	−11.86	0.7550	0.3105	0.2450	0.6895	0.6712	0.4204	0.5458	15
5	−41.60	−15.59	0.3673	0.0921	0.6327	0.9079	0.4414	0.3551	0.3983	25
6	−36.06	−10.11	0.9539	0.4126	0.0461	0.5874	0.9156	0.4598	0.6877	7
7	−42.37	−16.29	0.2859	0.0512	0.7141	0.9488	0.4118	0.3451	0.3785	26
8	−40.28	−14.26	0.5074	0.1696	0.4926	0.8304	0.5037	0.3758	0.4398	22
9	−39.07	−13.12	0.6356	0.2366	0.3644	0.7634	0.5784	0.3958	0.4871	18
10	−43.21	−7.58	0.1971	0.5604	0.8029	0.4396	0.3838	0.5321	0.4579	19
11	−44.02	−8.46	0.1120	0.5093	0.8880	0.4907	0.3602	0.5047	0.4325	23
12	−38.85	−5.08	0.6592	0.7071	0.3408	0.2929	0.5947	0.6306	0.6126	11
13	−38.00	−2.37	0.7488	0.8655	0.2512	0.1345	0.6656	0.7880	0.7268	4
14	−38.03	−0.71	0.7454	0.9626	0.2546	0.0374	0.6626	0.9305	0.7966	1
15	−37.89	−2.40	0.7603	0.8639	0.2397	0.1361	0.6759	0.7861	0.7310	3
16	−38.43	−2.81	0.7027	0.8399	0.2973	0.1601	0.6271	0.7575	0.6923	6
17	−42.32	−6.76	0.2912	0.6084	0.7088	0.3916	0.4136	0.5608	0.4872	17
18	−38.47	−7.52	0.6985	0.5640	0.3015	0.4360	0.6239	0.5342	0.5790	13
19	−45.07	−6.95	0.0000	0.5976	1.0000	0.4024	0.3333	0.5541	0.4437	20
20	−39.80	−1.74	0.5585	0.9025	0.4415	0.0975	0.5311	0.8368	0.6839	8
21	−39.69	−2.75	0.5699	0.8433	0.4301	0.1567	0.5376	0.7614	0.6495	9
22	−39.47	−1.35	0.5927	0.9254	0.4073	0.0746	0.5511	0.8701	0.7106	5
23	−41.43	−3.38	0.3854	0.8067	0.6146	0.1933	0.4486	0.7212	0.5849	12
24	−38.46	−0.47	0.7003	0.9770	0.2997	0.0230	0.6252	0.9560	0.7906	2
25	−42.17	−4.04	0.3078	0.7678	0.6922	0.2322	0.4194	0.6829	0.5511	14
26	−43.22	−5.16	0.1968	0.7024	0.8032	0.2976	0.3837	0.6269	0.5053	16
27	−38.18	−4.86	0.7298	0.7199	0.2702	0.2801	0.6492	0.6409	0.6450	10

**Table 4:** Analysis of Variance for Transformed Response (Erosion Rate).

Source	DF	Seq SS	Contribution	Adj SS	Adj MS	F-Value	*p*-Value
Velocity m/s	2	21,583	3.39%	21,583	10,791	0.81	0.463
Angle of Incident (Deg)	2	302,433	47.51%	175,089	87,545	6.55	0.008
Crumb Rubber (%)	2	222,671	34.98%	158,993	79,496	5.95	0.012
Stand of Distance (mm)	2	4830	0.76%	4830	2415	0.18	0.836
Erodent size (Mic)	2	62,308	9.79%	62,308	31,154	2.33	0.129
Error	16	22,789	3.58%	213,765	13,360		
Total	26	636,568	100.00%			R^2^	96.42%

**Table 5:** Analysis of Variance for Transformed Response (Erosion Efficiency).

Source	DF	Seq SS	Contribution	Adj SS	Adj MS	F-Value	*p*-Value
Velocity m/s	2	1980.65	82.28%	1980.65	990.324	79.99	0.000
Angle of Incident (Deg)	2	116.88	4.86%	116.88	58.442	4.72	0.024
Crumb Rubber (%)	2	51.30	2.13%	51.30	25.649	2.07	0.158
Standoff Distance (mm)	2	53.11	2.21%	53.11	26.557	2.15	0.150
Erodent size (Mic)	2	7.16	0.30%	7.16	3.581	0.29	0.753
Error	16	198.08	8.23%	198.08	12.380		
Total	26	2407.19	100.00%			R^2^	91.77%

**Table 6:** Results of confirmation experiment.

	Random	Optimal Parameters	
		Predicted	Experimental
Combination Level	A_2_B_3_C_2_D_2_E_2_	A_2_B_2_C_3_D_3_E_3_	A_2_B_2_C_3_D_3_E_3_
Erosion Rate	821.14		410.2
Erosion efficiency	13.759		5.239
GRG	0.4325	0.746	0.8353

## Data Availability

All the required data is available within the article.
